# A nurse‐led intervention in patients with newly diagnosed cancer and Type 2 diabetes: A pilot randomized controlled trial feasibility study

**DOI:** 10.1002/cam4.6118

**Published:** 2023-05-22

**Authors:** Lisa Scarton, Tarah Nelson, Ara Jo, LaToya J. O’Neal, Yingwei Yao, Shavondra Huggins, Anatolia B. Legaspi, Mariah J. McClaren, Jake S. Cabassa, Joan M. Burgos Melendez, Juan M. Munoz‐Pena, Merry J. Markham, Martina C. Murphy, Jonathan A. Chatzkel, Sherise Rogers, Thomas J. George

**Affiliations:** ^1^ College of Nursing University of Florida Gainesville Florida USA; ^2^ College of Public Health and Health Professions University of Florida Gainesville Florida USA; ^3^ College of Agricultural and Life Sciences, Institute of Food and Agricultural Sciences University of Florida Gainesville Florida USA; ^4^ College of Pharmacy University of Florida Gainesville Florida USA; ^5^ Division of Endocrinology, Diabetes and Metabolism University of Florida College of Medicine Gainesville Florida USA; ^6^ Division of Hematology and Oncology University of Florida, College of Medicine Gainesville Florida USA

**Keywords:** clinical management, clinical trials, community outreach, medical oncology, screening

## Abstract

**Background:**

Undiagnosed Type 2 diabetes (T2D) has been associated with advanced stage cancer at diagnosis, higher mortality, and lower long‐term all‐cause survival. This was a RCT pilot study to examine the feasibility of a nurse‐led T2D intervention for adults with newly diagnosed cancer (≤3 months), and T2D, undiagnosed or untreated with medication, conducted at an outpatient oncology clinic affiliated with a large academic institution.

**Methods:**

Participants needed to meet the eligibility criteria including a HbA1c level between 6.5% and 9.9%. Randomization was 1:1 to a 3‐month intervention that consisted of nursing‐led diabetes education and immediate initiation of metformin versus referral to primary care for usual care (control).

**Results:**

Three hundred and seventy nine patients were screened using EHR, 55 agreed to participate, and 3 had eligible HbA1c levels and were randomized in the study. Primary reasons for study exclusion included life expectancy ≤2 years (16.9%), current use or inability to tolerate metformin (14.8%), and abnormal labs that contraindicated metformin use (13.9%).

**Conclusion:**

This study was not feasible due to recruitment inefficiencies, but acceptable to all who qualified.

## Graphical abstract

Undiagnosed Type 2 diabetes (T2D) has been associated with advanced stage cancer at diagnosis, higher mortality, and lower long‐term all‐cause survival. This was a pilot RCT study to examine the feasibility of a nurse‐led T2D intervention for adults with newly diagnosed cancer (=3 months), and T2D, undiagnosed or untreated with medication, conducted at an outpatient oncology clinic affiliated with a large academic institution. This study was not feasible due to the cost, time, and personnel needed to screen participants with a point‐of‐care HbA1c test.

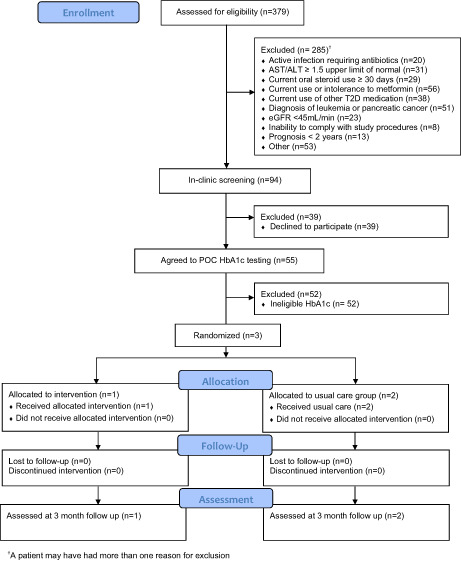

## 1 BACKGROUND

Cancer and diabetes are highly prevalent in the United States.^1^, ^2^ Undiagnosed or poorly managed Type 2 diabetes (T2D) has been associated with advanced‐stage cancer at diagnosis, higher mortality, and lower long‐term all‐cause survival.^3^, ^4^, ^5^ Patients with both T2D and cancer may face challenges with polypharmacy, treatment complications, and poor glycemic control. It is imperative to detect and manage T2D in patients with newly diagnosed cancer.

Nurses are uniquely positioned to fill critical gaps between T2D and cancer care. A nurse‐led intervention using first‐line treatment for T2D may improve the current standard of care to detect and manage undiagnosed T2D in newly diagnosed cancer patients.^6^ Metformin is an ideal medication for patients with newly diagnosed cancer and T2D because it improves insulin resistance, inhibits cell proliferation, reduces colony formation, and causes partial cell cycle arrest in cancer cell lines.^7^ The aims of this study were to examine the feasibility of a nurse‐led intervention comprised of brief T2D management education with metformin initiation for adults with newly diagnosed cancer (≤3 months) and newly diagnosed or untreated T2D as well as to examine change in HbA1c levels after the 3‐month intervention.

## 2 METHODS

### 2.1 Study design and population

This was a pilot, parallel‐group randomized controlled trial (RCT) feasibility study in adults with newly diagnosed cancer and undiagnosed or untreated T2D to test a nurse‐led 3‐month intervention for managing T2D. The study took place from November 2020 to November 2021, with a pause due to COVID‐19 restrictions and research assistant (RA) training from December 2020 to April 2021. The extensive RA training was conducted by the primary investigator, a nurse with expertise in T2D, and consisted of, among other topics, informed consent, T2D education, and point‐of‐care (POC) HbA1c testing based upon study protocol. Patients were first prescreened using the electronic health record (EHR) and then screened in‐clinic at an academic‐affiliated outpatient oncology clinic We planned to screen approximately 800 adults and recruit a sample of up to 40 with a goal of retaining 32 subjects (≥80%). This sample size would have sufficient power (80%) to detect a Type I error of 0.05 with an effect size of 1. No interim analysis was planned.

Participants meeting the prescreening criteria (Table S1) completed an informed consent, and a RA collected a POC HbA1c test. If HbA1c was ≥6.5%, a second test was performed to confirm diagnosis.^8^ Participants with HbA1c <6.5% or >9.9% were ineligible. The latter were referred to their primary care provider (PCP) or endocrinologist for T2D care. Participants meeting all eligibility criteria completed a second consent and were assigned to either the nurse‐led intervention or usual care group using stratified (HbA1c < 8% vs. ≥8%) block randomization with a block size of 4. Randomization sequence was generated using R statistical software. POC HbA1c testing was repeated for all participants at or near their 3‐month oncology follow‐up visit.

The nurse‐led 3‐month intervention consisted of diabetes education and standard metformin initiation. Participants received information on how to take metformin using a standardized titration protocol, potential side effects of metformin, and how to measure their blood glucose levels using a glucometer. Prescriptions for both metformin as well as a blood glucose testing kit were given to participants. The oncology visit note, HbA1c testing results, and study enrollment status were relayed to the participant's PCP. Participants were contacted by a nurse weekly by phone for the first month and then once monthly for 2 months to monitor metformin use, potential side effects, and answer any questions. Phone calls lasted approximately 15 min. Participants were also asked for a pill count to monitor medication adherence and their blood glucose logbook was reviewed.

Participants in the usual care group were referred to a PCP for T2D management. The oncology visit note and HbA1c testing results were relayed to the participant's PCP. Participants were contacted weekly by phone for the first month and then once monthly for 2 months to discuss their status. The trial was registered in clinicaltrials.gov (ID: NCT04468243) and IRB approval was obtained from the University of Florida (IRB# IRB201902145).

### 2.2 Feasibility metrics

Recruitment efficiency, retention rates, acceptability, and diabetes self‐management were assessed. At the 3‐month follow‐up, all participants completed a valid and reliable study acceptability scale that included topics such as (1) screening process; (2) time commitment; (3) education content; and (4) patient satisfaction (scores >8 indicated adequate acceptability).^9^ The Summary of Diabetes Self‐Care Activities Assessment, a brief valid and reliable self‐report questionnaire measuring the frequency of diabetes self‐care activities performed in the previous week, was given to participants at baseline and the 3‐month follow‐up.^10^ Domains included diet (items 1–4), exercise (items 5 and 6), blood sugar (items 7 and 8), foot care (items 9 and 10), and smoking status (items 11 and 12).

### 2.3 Statistical analysis

The data, stored in the REDCap database, were exported to R statistical software for analysis. R studio version (2022.02.2 + 485) was used to calculate descriptive statistics for feasibility metrics (frequency, percentage, mean) and sociodemographic characteristics of screened patients. Pretest and posttest HbA1c levels were reported. Fisher's exact tests were performed to compare characteristics between participants that agreed to in‐clinic screening versus those that declined.

## 3 RESULTS

### 3.1 Participants

A total of 379 patients were screened using the EHR, 94 patients met criteria and were approached in clinic with 59% (55/94) agreeing to POC HbA1c testing. Primary reasons for study exclusion during prescreening included life expectancy ≤2 years and patients diagnosed with leukemia or pancreatic cancer (16.9%), current use or inability to tolerate metformin (14.8%), and abnormal labs that contraindicated metformin use (eGFR, AST/ALT) (13.9%). No significant demographic differences were noted between patients that agreed versus patients that declined to participate (Table 1). Additional sociodemographic data were obtained for those that agreed to participate (Table S2). Three participants had eligible HbA1c levels and were enrolled in the study. Of the three participants, one was non‐Hispanic Black and the other two were non‐Hispanic White with ages ranging from 49 to 74 years. Cancer types included Stages 2–4 breast and colorectal cancer with treatment consisting of either chemotherapy alone or chemotherapy and surgery.

**TABLE 1:** Baseline characteristics among patients screened in clinic.

*N* (%)			*p*‐value^a^
Characteristic	Agreed (*n* = 55)	Declined (*n* = 39)	
Sex			
Male	20 (36)	16 (41)	0.67
Female	35 (64)	23 (59)	
Race			
White	40 (73)	32 (82)	0.45
Black	6 (11)	4 (10)	
Asian	4 (7)	0 (0)	
Native Hawaiian or Pacific Islander	1 (2)	0 (0)	
American Indian	0 (0)	0 (0)	
Other	1 (2)	2 (5)	
Decline	3 (5)	1 (3)	
Ethnicity			0.31
Hispanic or Latino	1 (2)	3 (8)	
Not Hispanic or Latino	51 (93)	35 (90)	
Decline to report	3 (5)	1 (2)	
Age			
18–44	9 (16)	3 (8)	0.46
45–64	23 (42)	22 (56)	
65–74	18 (33)	12 (31)	
75–84	5 (9)	2 (5)	
Cancer type			
Breast cancer	16 (29)	8 (21)	0.92
Colorectal cancer	7 (13)	7 (18)	
Female reproductive/gynecologic cancer	4 (7)	3 (8)	
Head and neck cancer	6 (11)	4 (10)	
Lymphoma	7 (13)	4 (10)	
Lung cancer	7 (13)	6 (15)	
Male reproductive cancer	1 (2)	1 (3)	
Miscellaneous gastrointestinal cancer	3 (5)	3 (8)	
Neuroendocrine cancer	0 (0)	1 (3)	
Sarcoma	0 (0)	1 (3)	
Urinary cancer	4 (7)	1 (3)	
Cancer treatment			
Chemotherapy only	18 (33)	8 (21)	0.50
Chemotherapy/radiation	5 (9)	5 (13)	
Chemotherapy/surgery	8 (15)	8 (21)	
Chemotherapy/surgery/radiation	5 (9)	6 (15)	
Surgery/radiation	3 (6)	2 (5)	
Surgery only	4 (7)	0 (0)	
Radiation only	3 (6)	1 (3)	
No treatment	9 (16)	9 (23)	

^a^ *p*‐Values were based on Fisher's exact test.

### 3.2 Feasibility metrics

**FIGURE 1:**
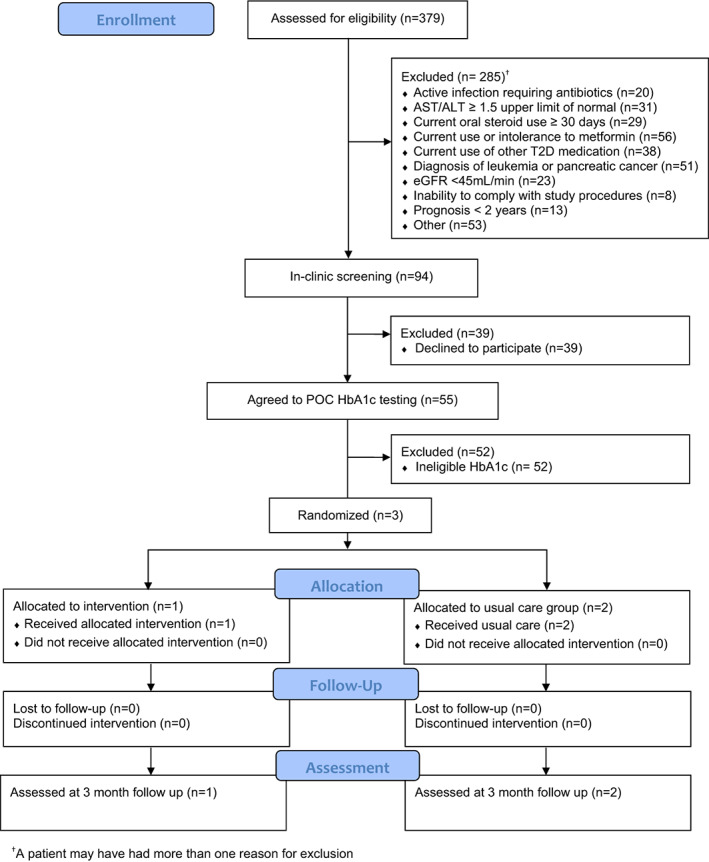
CONSORT diagram.

*Recruitment efficiency*. While the sample population was easily accessible and identifiable through EHR review, there was a lack of recruitment efficiency during the screening process. 75% (285/379) of patients were excluded prior to in‐clinic screening (Figure 1).

*Retention rates*. No participants (*N* = 3) were lost to follow‐up.

*Acceptability*. All participants scored this study “acceptable,” reporting they enjoyed the study, found it was the right length, and thought it would be well received by other patients. All participants also reported the diabetes education and follow‐up phone calls were helpful.

*Diabetes self‐management*. From baseline to 3‐month follow‐up, the intervention group (*n* = 1) had a score improvement in the blood glucose testing (difference = 13) and exercise domain (difference = 4), no difference in the foot care domain and smoking status (the participant was a nonsmoker), and a decrease in the diet domain (difference = −3). The participants in the usual care group (*n* = 2) had an improvement in the blood sugar testing domain (mean difference = 3.5), diet domain (mean difference = 2), and a decrease in the foot care (mean difference = −1) and exercise domains (mean difference = −7). Smoking status decreased (1–10 to 0 cigarettes) at the 3‐month follow‐up for one participant in the usual care group while there was no change for the second participant.

### 3.3 HbA1c levels

After 3 months, the HbA1c level of the intervention group participant (*n* = 1) decreased from 6.7% to 5.1% and the HbA1c level for the usual care group participants (*n* = 2) decreased from 7.2% and 6.7% to 5.5% and 5.9%, respectively. The intervention group participant received prompt diabetes education and started metformin immediately, while the usual care participants started metformin 60 and 90 days later, respectively.

## 4 DISCUSSION

This pilot study was closed due to failing feasibility metrics. The largest barrier was identifying eligible patients with POC HbA1c determinations. One way to improve identification of future patients would be to integrate HbA1c testing into order sets for all newly diagnosed cancer patients entering the clinic and broaden the eligibility criteria to a more pragmatic assessment of clinical acceptability for metformin use, rather than only including those with laboratory values within normal limits. This area of research is important because undiagnosed diabetes prior to cancer diagnosis may deteriorate blood glucose level and cause poor prognosis due to difficulty of co‐managing both comorbidity and low healthcare provider contact.^11^, ^12^ It is also associated with higher mortality.^3^ Further, a recent Korean national cohort study found that a diagnosis of cancer elevated the risk of diabetes, independent of known risk factors for diabetes.^13^

Some patients have difficulty prioritizing primary care while undergoing cancer treatment and PCPs may also defer treatment of chronic conditions during this time.^12^ In a recently published metanalysis, researchers found that nurse‐led titration of glucose‐lowering medications was associated with improved HbA1c levels and patient satisfaction.^14^ Therefore, a nurse‐led intervention within oncology clinics to detect and initiate early treatment for diabetes has the potential to improve care. Moreover, there are known barriers to utilizing primary care for vulnerable populations, many at increased risk of both cancer and diabetes.^2^, ^15^, ^16^ While the intervention group (*n* = 1) started metformin 2–3 months earlier than the usual care group, HbA1c levels improved in both groups and all patients. A potential reason why HbA1c levels in the usual care group may have improved may be social desirability bias resulting from weekly or monthly nurse check‐in phone calls that may have inadvertently served as a reminder to schedule an appointment with their PCP to discuss T2D. In addition, all three participants lost 5%–10% of their body weight, likely related to their cancer diagnosis or treatment, which may have impacted their HbA1c levels during the study period. We remain optimistic that implementation of a nurse‐led intervention has the potential to improve the early detection and management of undiagnosed T2D among those newly diagnosed with cancer.

A limitation of this study was the small number of eligible patients. While many patients were agreeable to participate, the time needed to screen patients for T2D using POC HbA1c testing was inefficient. Participation may have been affected by the time and burden of a fingerstick blood sample within their first oncology visit. This study was also conducted during the COVID‐19 pandemic which may have had a negative effect on participation. Due to low enrollment, we were unable to make conclusions regarding the HbA1c levels, diabetes self‐management, or participant acceptability of the study.

This study was strengthened by the large collaborative, interdisciplinary team. Another strength of this study was T2D screening within their first oncology visit and rapid access to T2D education, glucometer, and treatment with metformin for patients newly diagnosed with cancer without having to coordinate care with their primary providers. In an effort to improve the noted inefficiencies and enrollment, it is recommended that future researchers include HbA1c testing with existing order sets for all newly diagnosed cancer patients entering the clinic and consider assessing and expanding eligibility criteria as appropriate. Also, decreased access to health care is associated with undiagnosed T2D, uncontrolled T2D, and cancer diagnosed at an advanced stage so it is imperative that underserved populations at increased risk of experiencing challenges accessing health care are included in future studies.^16^, ^17^, ^18^

## 5 CONCLUSIONS

Despite a number of patients being interested in participating, only three were enrolled in this study. This study was not feasible due to the cost, time, and personnel needed to screen participants with a POC HbA1c test. This suggests a willingness and interest for patients to participate, but reliance on POC HbA1c testing is inefficient. While findings were promising, we are unable to make any meaningful conclusions regarding the HbA1c levels, diabetes self‐management, or participant acceptability of the study. However, routine HbA1c screening for all patients newly diagnosed with cancer could identify patients in whom T2D management is indicated. With refinements to this intervention, there remains potential for improving access to T2D care as well as detection and management of undiagnosed T2D for those newly diagnosed with cancer with opportunities for early and safe intervention through nursing led point‐of‐care T2D interventions.

## AUTHOR CONTRIBUTIONS

**Lisa Scarton:** Conceptualization (equal); investigation (equal); project administration (equal); writing – original draft (lead). **Tarah Nelson:** Data curation (equal); formal analysis (lead); writing – original draft (lead). **Ara Jo:** Conceptualization (equal); writing – review and editing (equal). **LaToya J O’Neal:** Conceptualization (equal); writing – review and editing (equal). **Yingwei Yao:** Conceptualization (equal); formal analysis (supporting); writing – review and editing (equal). **Shavondra Huggins:** Resources (equal); writing – review and editing (equal). **Anatolia B. Legaspi:** Data curation (equal); investigation (equal); project administration (equal); writing – review and editing (equal). **Mariah J. McClaren:** Investigation (equal); writing – review and editing (equal). **Jake S. Cabassa:** Investigation (equal); writing – review and editing (equal). **Joan M. Burgos Melendez:** Investigation (equal); writing – review and editing (equal). **Juan Manual Munoz Pena:** Supervision (equal); writing – review and editing (equal). **Merry J. Markham:** Supervision (equal); writing – review and editing (equal). **Martina C. Murphy:** Supervision (equal); writing – review and editing (equal). **Jonathan A. Chatzkel:** Supervision (equal); writing – review and editing (equal). **Sherise C. Rogers:** Supervision (equal); writing – review and editing (equal). **Thomas J George:** Conceptualization (equal); supervision (equal); writing – review and editing (equal).

## FUNDING INFORMATION

Support was provided by the University of Florida Cancer Center pilot grant CPS‐FY20‐01 and Grant Numbers U54CA233444, U54CA233396, and U54CA233465 from the National Institutes of Health (NIH), National Cancer Institute (NCI). The content is solely the responsibility of the authors and does not necessarily represent the official views of the NIH or NCI.

## CONFLICT OF INTEREST STATEMENT

No potential conflicts of interest relevant to this article were reported.

## ETHICS STATEMENT

All participants completed and signed an informed consent form prior to completing the in‐clinic point‐of‐care (POC) HbA1c test. If enrolled in the study, participants then completed and signed a second consent. This pilot study was approved by the IRB of the University of Florida, IRB # IRB201902145.

## Supporting information


**Table S1.** Table S2.

## Data Availability

The data generated from this study are available from the corresponding author upon reasonable request.
