# Supplementary material for: A nurse‐led intervention in patients with newly diagnosed cancer and Type 2 diabetes: A pilot randomized controlled trial feasibility study
Source: Cancer Med. 2023 May 22;12(11):12874–80. doi: 10.1002/cam4.6118 (PMC10278510; doi:10.1002/cam4.6118)
Supplement: Supplementary file 1 — Table S1. Table S2. [file CAM4-12-12874-s001.docx]

Supplementary Information

| **Table S1. Inclusion and Exclusion criteria** | |
| --- | --- |
| **Inclusion criteria assessed during pre-screening** | **Exclusion criteria assessed during pre-screening** |
| 18 years or older | Diagnosis of leukemia or pancreatic cancer as the new cancer diagnosis |
| Using pathology or other available medical reports, a new diagnosis of a solid tumor cancer or lymphoma within the past 3 months | Most current eGFR <45mL/minute/1.73m^2^ |
| Patients being treated with curative intent or those with a prognosis estimated to be >2 years, as best assessed by the oncologist | Most current liver enzyme values (AST and ALT) over 1.5 times the upper limit of normal (or over 5 times the upper limit of normal if patient has liver metastases). |
| First medical oncology outpatient visit within the past 3 months | Clinical sequelae of advanced cirrhosis as documented by the treating oncologist or advanced practice providers |
| Speaks and reads English | Active infection requiring systemic antibiotics |
| Can comply with study related procedures, per the treating oncologist or advanced practice provider | Taking continuous systemic steroids for 30 days or more at time of enrollment. Note, intermittent doses of steroids (e.g., for chemotherapy nausea), or inhaled, topical, and eye drop steroids are not exclusion criteria |
| Plans to continue cancer care facility | Enrolled in another study with similar outcomes |
|  | Being treated with medication for T2D |
|  | Known inability to tolerate metformin |
| **Additional inclusion criteria at point of care** | **Additional exclusion criteria at point of care** |
| HbA1c level between 6.5% and 9.9% | HbA1c level <6.5% or >9.9% |

| **Table S2. Additional baseline characteristics among patients who agreed to participate** | |  |  |
| --- | --- | --- | --- |
| **Characteristic** | **Agreed** (N = 55) No. (%) | |  |
| **Employment**^†^ |  | |  |
| Employed full-time | 11 (20) | |  |
| Employed part-time | 4 (7) | |  |
| Homemaker | 2 (4) | |  |
| Retired | 21 (39) | |  |
| Unemployed | 12 (22) | |  |
| Other | 4 (7) | |  |
| **Income**^†^ |  | |  |
| Comfortable | 29 (54) | |  |
| Do not have enough to make ends meet | 5 (9) | |  |
| Just enough to make ends meet | 20 (37) | |  |
| **Educational Status**^†^ |  | |  |
| Doctoral Degree | 3 (6) | |  |
| Master's Degree | 3 (6) | |  |
| Bachelor's degree | 11 (20) | |  |
| Associate Degree | 8 (15) | |  |
| High School | 26 (48) | |  |
| Less than High School | 3 (6) | |  |
| **Marital Status**^†^ |  | |  |
| Married/Partnered | 27 (50) | |  |
| Single | 13 (24) | |  |
| Divorced/Separated | 8 (15) | |  |
| Widowed | 6 (11) | |  |
| ^†^1 participants’ data were missing |  |  | |
